# Prospective biomarkers of posttraumatic stress disorder in children and adolescents: a systematic review and meta-analysis

**DOI:** 10.1038/s41398-026-03939-1

**Published:** 2026-03-30

**Authors:** T. H. Sharp, M. Bailey, C. Burke, A. Giuliani, L. V. Hiscox, E. Alisic, R. Kumsta, S. Seedat, S. L. Halligan

**Affiliations:** 1 Department of Psychology, University of Bath, Bath, UK; 2 Population Health Sciences, Bristol Medical School, University of Bristol, Bristol, UK; 3 Cardiff University Brain Research Imaging Centre (CUBRIC), School of Psychology, Cardiff University, Cardiff, UK; 4 Melbourne School of Population and Global Health, University of Melbourne, Carlton, VIC Australia; 5 Department of Behavioural and Cognitive Sciences, Faculty of Humanities, Education and Social Sciences, University of Luxemburg, Esch-sur-Alzette, Luxemburg; 6 South African Medical Research Council Unit on the Genomics of Brain Disorders, Department of Psychiatry, Faculty of Medicine and Health Sciences, Stellenbosch University, Cape Town, South Africa

**Keywords:** Predictive markers, Psychiatric disorders

## Abstract

Trauma exposure in children and young people (CYP) can lead to the development of posttraumatic stress disorder (PTSD). Identification of biomarkers prospectively associated with PTSD can provide critical insight into the mechanisms underpinning this disorder and potentially aid in identifying CYP vulnerable to persistent symptoms. A systematic search of databases was conducted up until February 2024 to identify studies testing associations of prospective biomarkers with PTSD outcomes in CYP. A narrative synthesis of study characteristics, quality, and findings was conducted. Meta-analysis using a random-effects model was performed when two or more comparable studies were identified. Searches yielded 2039 articles, with 283 identified as relevant after title/abstract screening. Twenty-one studies met eligibility criteria in the following biological domains: hormonal, immunological, cardiovascular, and multisystem. The majority of studies focussed on naturalistic recovery of CYP exposed to acute trauma. Significant heterogeneity was observed, including in trauma type, selection, and biomarker measurement. Across biomarkers, relatively consistent evidence was observed for a prospective association between elevated heart rate post-trauma and increased risk of PTSD persistence only, with limited evidence in other domains. Our review highlights a limited evidence base for prospective biomarkers of the development of PTSD in CYP, with methodological issues limiting inferences that can be drawn. A notable lack of evidence from low-and-middle income countries, despite chronic and severe trauma being endemic in these settings, was identified. Further research using standardised protocols from large, representative samples will be critical in the identification of biomarkers of PTSD risk and resilience.

## Introduction

Children and young people exposed to trauma are vulnerable to developing posttraumatic stress disorder (PTSD), which can present a significant threat to a young person’s developmental trajectory [1]. Up to 20% of children and young people(CYP) will develop PTSD post-trauma [2], with the first six months identified as a critical window during which symptoms may spontaneously remit or become persistent and require intervention [3]. Leading hypotheses propose that development of persistent PTSD in CYP may be partially driven by biological changes post-trauma and/or by pre-existing biological vulnerabilities that influence post-trauma symptom severity and persistence [4]. A causal understanding of the biological factors that are associated with the onset and/or progression of PTSD is critical to informing; 1) our ability to identify CYP who are particularly vulnerable to persistent symptoms, 2) our understanding of mechanisms by which risk is conferred, and 3) for identification of potential therapeutic targets to allow for precision treatment of PTSD.

In adults, there is substantial evidence that a PTSD diagnosis is associated with differences in biological markers when compared to healthy or trauma-exposed controls, with alterations reported across stress-related biological systems of the limbic-hypothalamic-pituitary adrenal (LHPA) axis, autonomic nervous system (ANS), inflammatory response, gut microbiome, and underpinning neurological structures and functions [5–7]. LHPA, ANS and inflammatory activity have been widely studied, with meta-analyses providing evidence of cortisol hyposecretion [8, 9], increased autonomic activity/reactivity [10–12], and heightened inflammation [13] in association with adult PTSD. A role of gut dysbiosis has also been postulated, with emerging evidence that disruptions to the gut-brain axis may contribute to PTSD symptomology via impacts on inflammation, neurotransmitter signalling, and the stress response [14, 15]. Finally, epigenome-wide association studies have begun to reveal differences in DNA methylation profiles in individuals with PTSD compared to controls, including DNA methylation differences in genes with roles in immune response and brain function [16]. Beyond these cross-sectional findings, a smaller but growing body of prospective work suggests some biological alterations are involved in aetiology. Longitudinal studies in trauma-exposed adult cohorts have shown that elevated baseline inflammatory markers such as C-reactive protein (CRP) are associated with increased risk of subsequent PTSD [17]. Lower cortisol levels in the acute phase post-trauma have also been linked to increased PTSD risk [18], though the strength and direction of effect is not consistent across studies [12].

It is important to note that for a biomarker to be of clinical use, it must 1) show consistent and replicable associations with a clinical outcome across independent studies, 2) be biologically plausible based on mechanistic understanding, and 3) demonstrate some degree of diagnostic, prognostic, or therapeutic utility [19]. In adult populations, PTSD biomarkers such as differences in cortisol levels, elevated pro-inflammatory cytokines, and increased autonomic reactivity meet the first two of these criteria, supported by the body of evidence described above, including replication in diverse populations, meta-analysis and experimental findings. However, their translation into clinical practice remains limited, and they have not yet shown added value relative to existing tools for clinical decision-making in PTSD.

Despite these advances in the adult PTSD field, our understanding of the psychobiology of PTSD among CYP is particularly limited. Given the profound neurobiological and physiological changes occurring across childhood and adolescence, it cannot be assumed that biomarker differences identified in adults generalise to CYP. This represents a critical research gap, as epidemiological studies have identified striking increases in the prevalence of PTSD across adolescence, making this a critical period in which to identify biological markers earlier in the course of progression [20]. Major questions remain relating to which biological factors are causal in the aetiology of youth PTSD, with the limited available evidence in this group often diverging from observations in adults [12, 21, 22]. In contrast to adult findings, higher circulating cortisol levels have been shown to be prospectively associated with PTSD development in CYP [23]. A recent meta-analysis has extended this finding, demonstrating that participant age moderated the relationship between cortisol and PTSD risk, such that cortisol was positively associated with PTSD risk in CYP, and negatively associated with PTSD risk in adults. In addition, the authors found suggestive evidence for a similar moderation effect for resting heart rate and systolic blood pressure [12]. Therefore, in this systematic review we aimed to synthesise and evaluate the evidence regarding the association of prospective biological markers with onset, progression and/or recovery from PTSD in CYP, providing an overview of peripheral biomarkers that represent viable targets for prediction and/or therapeutic interventions.

## Methods

We used the Preferred Reporting Items for Systematic Reviews and Meta-Analyses statement (Supplementary Table 1) [24], and registered our protocol on the PROSPERO database (CRD42022377785). Covidence software (https://www.covidence.org/) was used to facilitate screening and extraction.

### Search process and study selection

We performed literature searches between November and December 2022, and a search update in February 2024. Articles were identified by searching databases MEDLINE, Embase, and PsycINFO using the Ovid interface. Hand searches of the reference lists of included studies and reviews were conducted. The search included terms related to; a) PTSD and PTSS (posttraumatic symptom severity), b) CYP, and c) biological and physiological markers. Terms related to neural biomarkers were included in the search strategy, but neuroimaging studies were not included in the present synthesis, which is restricted to peripheral biomarkers only (hormonal, immunological, cardiovascular, and multisystem measures). Eligible studies were English-language, peer-reviewed articles in human samples with a mean age ≤18 years, using a prospective design in which a biomarker marker was assessed before PTSD/PTSS. Studies using a pseudo-longitudinal design (e.g., those using hair samples which capture cumulative data over time), were eligible. Full details can be found in Supplementary Methods.

Co-authors THS, CB, and LH screened titles and abstracts. Duplicate articles were manually removed. A random selection of articles (20%, n = 371) were independently screened by a second reviewer, with a high level of reliability observed (100%). All articles eligible for full text review were screened independently by two reviewers, with discrepancies (N = 5) resolved via discussion with SLH.

### Data extraction

Co-authors THS, CB, LH, AG, and MB extracted study data comprising: design, population characteristics, biomarker, methods used to ascertain exposures, timepoints of PTSD measurement, method of ascertainment of PTSD outcome, study findings (from primary analysis, with data from most adjusted model extracted) and control of covariates. Study quality was assessed using an adapted version of the Newcastle–Ottawa Scale (NOS) for cohort studies [25, 26]. Adapted items and scoring system are detailed in Supplementary Methods and Supplementary Table 2.

### Strategy for data synthesis

We completed a narrative synthesis of study characteristics and findings, by biological domain. We captured sample characteristics, trauma exposure, measure of PTSD, key study outcomes, effect sizes, confidence intervals, and p-values reported by each individual study. Significant heterogeneity between studies was anticipated, and therefore meta-analysis was conducted for if study methodology, exposure/outcome definition, and reporting of effect measures/associations were consistent. We identified a sufficient degree of homogeneity across studies assessing the association of HR in the acute phase posttrauma with PTSD outcomes (N = 7). In line with a previous meta-analysis in CYP [27], we restricted the analysis to bivariate correlations between acute-phase HR and PTSD outcomes. As a result, the pooled effect reflects unadjusted associations and may be biased by residual confounding. Meta-analysis was performed using the **metafor** R package (https://cran.r-project.org/web/packages/metafor/index.html). Full methodological details are provided in Supplementary Methods.

## Results

**Fig. 1:**
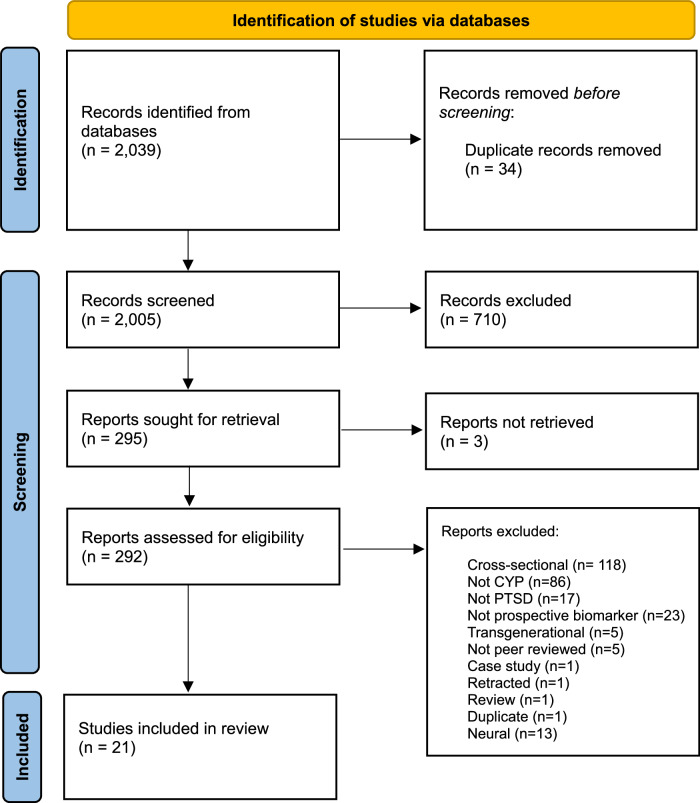
PRISMA flow diagram of systematic search for prospective biomarker studies of PTSD in CYP. PTSD (posttraumatic stress disorder), CYP (CYP and young people).

**Fig. 2:**
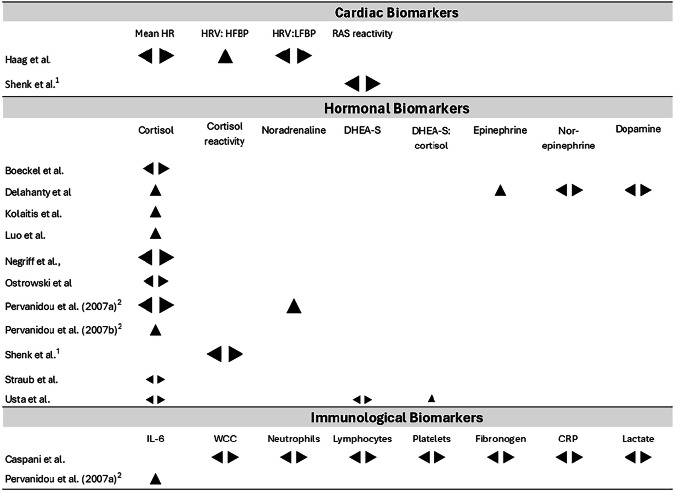
Effect direction plot depicting associations observed across studies. Association direction: upward arrow ▲= positive association, downward arrow ▼ = negative association, sideways arrow ◀ ▶ = null association. Total sample size: small arrow <50, medium arrow ≥50, large arrow ≥100.

In total, 2039 articles were imported into Covidence software for screening, with 34 duplicates removed. Of the remaining 2,005 articles, we identified 292 articles for full text review, which yielded 21 articles assessing biomarkers in the following domains: cardiac (n = 9), cardiac/neurohormonal (n = 2), hormonal (n = 9), immunological (n = 1), immunological/hormonal (n = 1) (see Fig. 1). No articles prospectively assessing the association of gastrointestinal, epigenomic, or metabolomic biomarkers with PTSD outcomes were identified. Study characteristics are summarised in Table 1. For studies where meta-analysis was not possible, associations from main analyses are displayed in an effect direction plot (Fig. 2). All extracted quantitative data can be found in supplementary tables S3-S5.

**Table 1:** Study details on all prospective studies subdivided by biomarker domain.

Biomarker	Author	Country	Study design	Mean Age at baseline (years)	Biomarker		PTSD Outcome	
					Measure	Source	Measure	Timepoint
Cardiac	*De Young* et al., [47]	Australia	Observational CYP hospitalised following accidental physical injury (n = 101) Female: 35%	10.8	HR (acute phase)	Extracted from medical records. Upon hospital arrival and within 24-h of admission.	Symptom severity (ADIS-C)	6 months
	*Bryant* et al., [45]	Australia	Observational CYP hospitalised following traumatic injury (n = 76) Female: 25%	PTSD: 7.63 pPTSD: 9.11 TD: 10.42	HR (acute phase)	Extracted from medical records. Within 24 h of hospital admission.	Diagnosis (PTSD-RI)	6 months
	*Olsson* et al., [49]	Australia	Observational CYP hospitalised following traumatic injury (n = 76) Female: 33%	10.81	HR (acute phase)	Extracted from medical records. Upon hospital arrival and within 24-h of admission.	Symptom severity (ADIS-C/P)	1 month 6 months
	*Nixon* et al., [50]	Australia	Observational CYP admitted to ED or paediatric inpatient ward (n = 48) Female: 35%	11.84	HR (acute phase)	Extracted from medical records. Upon hospital arrival.	Symptom severity (CPSS)	6 months
	*Nugent* et al., [51]	Ireland	Observational CYP admitted as trauma patients with GCS score >= 14 (n = 82) Female: 26%	13.21	HR (acute phase)	Extracted from medical records. During transport to hospital, hospital admission, and day of discharge.	Symptom severity (CAPS-CA)	6 weeks 6 months
	*Haag* et al., [55]	UK	Observational CYP admitted to ED after acute trauma (n = 132) Female: 39%	10.05	HR HRV	ECG data collected within 4 weeks of hospital admission.	Symptom severity (PTSD-RI)	3 months 6 months
	*Kassam-Adams* et al., [46]	US	Observational CYP admitted to hospital for RTA (n = 190) Female: 25%	11.2	HR (acute phase)	Extracted from medical records. Upon hospital arrival.	Full or partial diagnosis (CAPS-CA)	3 months
	*Zatzick* et al., [48]	US	Observational CYP admitted to hospital with intentional and unintentional injuries (n = 108) Female: 32%	15.9	HR (acute phase)	Extracted from medical records. Upon hospital arrival.	Symptom severity (PTSD-RI)	2 months 5 months 12 months
	*Marsac* et al., [52]	US	Observational CYP hospitalised for injury (n = 96) Female: 35%	10.6	HR (acute phase)	Extracted from medical records. Within 24 h of hospital admission.	Symptom severity (CPSS)	6 weeks 12 weeks
Cardiac and hormonal	*Nugent* et al., [43]	US	Observational CYP hospitalised for traumatic injury (n = 82) Female: 37%	13.25	HR Cortisol	Urine, fluorescent polarization immunoassay. Sampled over first 12 h of hospital admission.	Symptom severity (CAPS-CA)	6 weeks 6 months
	*Shenk* et al., [42]	US	Observational CYP exposed to maltreatment (n = 51), and unexposed comparison group (n = 59) Female: 100%	17.00	RSA and cortisol reactivity during stressor paradigm	Saliva, enzyme immunoassay. Within 12 months of reported maltreatment.	Symptom severity (CTI)	12 months
Hormonal	*Boeckel* et al., [32]	Brazil	Observational CYP exposed to IPV (n = 32) and unexposed comparison group (n = 27) Female: 50%	TE: 8.96 TE: 8.92	Cortisol	Hair, enzyme-linked immunosorbent assay. Maternal report of historical IPV.	Symptom severity (CPSS)	30 days
	*Luo* et al., [35]	China	Observational CYP with (n = 32) and without (n = 32) PTSD after 2008 Wenchuan earthquake and non-traumatized controls (n = 20) Female: 100%	PTSD:13.81 non-PTSD:13.84 TD:14.40	Cortisol	Hair, electrochemiluminescence immunoassay. Sample collected 7 months after event.	Diagnosis (CRIES and SCID)	7 months
	*Straub* et al., [40]	Germany	Observational CYP recruited from hospital who had experienced or witnessed an accident or emergency (n = 35) Female: 34%	10.71	Cortisol	Hair, immunoassay with chemiluminescence detection Sample collected within 16 days of event.	Symptom severity (CAPS-CA)	3 months
	*Pervanidou* et al., [38]	Greece	Observational CYP (n = 60) involved in MVA and comparison group (n = 40) Female: 33%	10.7	Noradrenaline Cortisol	Blood, liquid chromatography with electrochemical detection. Sample collected during hospital admission. Saliva and blood, electrochemiluminescence immunoassay. Sample collected during hospital admission.	Diagnosis (K-SADS-PL)	1 month 6 months
	*Kolaitis* et al., [34]	Greece	Observational CYP hospitalized for a road traffic accident (n = 60) Female: 32%	10.9	Cortisol	Saliva, electrochemiluminescence immunoassay. Sample collected during hospital admission.	Diagnosis (K-SADS-PL)	6 months
	*Usta* et al., [41]	Turkey	Quasi-experimental CYP with PTSD who had up to six sessions of EMDR therapy (n = 40) Female: 93%	15.4	Cortisol DHEA-S	Blood, chemiluminescence assay. Blood, chemiluminescence assay.	Treatment responder or non-responder (PTSD-RI and CGI)	6 weeks
	*Delahanty* et al., [33]	US	Observational CYP admitted to trauma centre (n = 82) Female: 32%	13.04	Cortisol Epinephrine Norepinephrine Dopamine	Urine, fluorescent polarization immunoassay. Sampled over first 12 h of hospital admission. Urine, reverse phase chromatography with electrochemical detection. Sampled over first 12 h of hospital admission.	Symptom severity (CAPS-CA)	6 weeks
	*Ostrowski* et al. [37]	US	Observational CYP admitted to trauma centre (n = 51) Female: 44%	13.35	Cortisol	Urine, fluorescent polarization immunoassay. Sampled over first 12 h of hospital admission.	Symptom severity (CAPS-CA)	6 weeks 7 months
	*Negriff* et al. [36]	US	Observational CYP with maltreatment history (n = 303) and comparison group (n = 151) Female: 47%	10.84	Cortisol during stressor paradigm.	Saliva, assay unreported. Samples collected within 1 month of exposure to maltreatment.	Symptom severity (YSSC)	12 months
Immunological	*Caspani* et al., [44]	UK	Observational CYP admitted to PICU for septic illness (n = 18), meningoencephalitis (n = 19) and other critical illnesses (n = 34) Female: 37%	9.54	WCC, lymphocytes, neutrophils CRP Platelets, fibrinogen Lactate	Blood, using nationally standardised procedures. Sample collected within 48 h of hospital admission.	Diagnosis, symptom severity (IES-8)	3-6 months
Immunological and hormonal	*Pervanidou* et al., [39]	Greece	Observational CYP involved in RTA (n = 48) and control group (n = 40) Female: 32%	10.7	Cortisol, IL-6	Saliva and blood, electrochemiluminescence immunoassay. Sample collected during hospital admission.	Symptom severity (K-SADS-PL)	1 month 6 months

## Hormonal

### Cortisol and DHEA-S

Ten studies explored the association between cortisol and subsequent PTSD or PTSS [28–39]. Of these, eight examined cortisol levels and subsequent PTSD/PTSS in a cohort in which all participants were exposed to trauma (primarily accidents and disasters) and the remainder compared a sample exposed to trauma to an unexposed control group.

Three studies examined hair cortisol, two with quasi-longitudinal designs in which hair samples captured cortisol for a period preceding PTSD measurement. *Luo* et al. examined hair cortisol levels in female adolescents with (n = 32) and without (n = 32) PTSD at 7-months following the 2008 Wenchuan earthquake, and in matched, non-exposed controls (n = 20) [31]. Hair samples were segmented to proxy cortisol secretion across four time windows capturing pre-, peri- and post-quake periods. Cortisol levels were higher in both trauma-exposed groups relative to controls in peri-earthquake segments; and in trauma-exposed subjects without PTSD compared to PTSD and/or control groups in segments spanning two to seven months post-quake. No PTSD specific effects were identified. *Boeckel* et al. examined cortisol levels in CYP exposed to intimate partner violence (IPV) (n = 32) and a comparison group (n = 27), with samples capturing cortisol in the month prior to assessment. No group differences in hair cortisol or correlations between hair cortisol levels and PTSS were observed. In the only true longitudinal study, *Straub* et al. completed clinical interviews and hair sampling at 5-days posttrauma with CYP who had been involved in an accident (n = 35), with follow-up interviews at 51 days [11]. Cortisol concentration in hair segments capturing the 3 months prior to trauma did not predict either acute stress disorder or PTSD symptom severity, though mean symptom scores were very low in this sample. Collectively, these studies do not provide evidence that hair cortisol levels are predictive of later PTSD symptoms in CYP.

Three studies examined urinary cortisol in CYP, all using samples from within the first 12 h of hospital admission post-injury, which may capture acute stress responses. *Ostrowski* et al. examined post-admission cortisol level in 54 injured CYP and found no associations between post-admission cortisol and PTSS measured at 6-weeks (n = 54) or at 7-months (n = 38) for the whole sample [33]. At 6-weeks, a positive correlation emerged only when n = 7 CYP with prior trauma were excluded from analyses. At 7-months there was weak evidence of a positive cortisol-PTSS association in boys in this subsample only. Using a similar design, *Delahanty* et al. found a positive association between post-admission urinary cortisol levels and PTSS at 6-weeks posttrauma among CYP with traumatic injuries [29]. The initial sample of 82 CYP tested in this study was reduced to n = 58 for analyses due to exclusions for prior mental illness, previous trauma exposure, or receiving steroids for injuries. PTSD prevalence was extremely low in both studies (n = 1) for the final assessment.

Three studies within the same sample examined basal salivary cortisol secretion among CYP recruited after hospitalisation for a road traffic accident (RTA) (n = 60), with biomarkers captured at baseline, 1 month, and 6 months later. *Pervanidou* et al. found salivary cortisol at baseline (captured via five timepoints throughout the day) to be elevated in injured CYP relative to a same-aged comparison group (n = 40). However, the authors did not find evidence to suggest cortisol predicted PTSD status at 1 or 6 months [34, 35]. In a follow-up analysis on the same sample, limited to evening cortisol samples only, *Kolaitis* et al. found raised evening cortisol levels at baseline to be associated with a higher risk of developing PTSD at 6 months, with a small effect size [30].

Two studies examined salivary cortisol in response to a stressor. *Negriff* et al. recruited CYP who had experienced maltreatment (n = 303) and a non-exposed comparison group (n = 151) [32]. Salivary cortisol response to a social stress test at baseline was examined in relation to self-reported PTSS at a 1-year follow-up, with analyses stratified by type of maltreatment, capturing sexual abuse, physical abuse, emotional abuse, and neglect in a hierarchical design. For the neglect group, a positive association between cortisol stress reactivity and 1-year PTSS was identified. *Shenk* et al. tested salivary cortisol reactivity to a stressor paradigm as a predictor of PTSS in a sample of female adolescents who had experienced maltreatment (n = 51) and an unexposed comparison group (n = 59). There was no evidence that cortisol reactivity at baseline predicted PTSS at a 1-year follow-up, though the stressor paradigm used did not reliably elevate cortisol in this sample.

*Usta* et al. examined whether fasting serum DHEA-S (dehydroepiandrosterone sulphate), cortisol, or DHEA-S: cortisol ratio predicted treatment response in adolescents with PTSD who had up to six sessions of EMDR (eye movement desensitization and reprocessing) therapy [37]. Participants were grouped based on response (n = 17) or non-response (n = 15), with response defined as a reduction in PTSD symptoms of >30%. Receiver Operating Characteristic (ROC) analysis was used to evaluate the diagnostic performance of the three biomarkers, identifying moderate power of DHEA-S: cortisol ratio to discriminate between treatment response and non-response. Cortisol and DHEA-S were not found to predict treatment response.

Taken together, these ten heterogeneous and relatively small studies do not provide consistent evidence that cortisol, across different sampling approaches, is a reliable prospective predictor of PTSD in CYP. Isolated positive associations tended to be confined to specific subgroups and time points, and effect sizes were small. The absence of a clear pattern should be interpreted cautiously, as studies differed substantially in biological metric, timing of collection relative to trauma and outcome, and control for diurnal variation, all of which may obscure underlying associations.

## Inflammatory markers

Two studies explored the association between peripheral inflammatory markers and subsequent PTSD or PTSS (posttraumatic stress symptoms) [35, 40]. *Caspani* et al. examined multiple blood inflammatory markers (i.e., white cell count, lymphocytes, neutrophils, CRP, fibrinogen and lactate) in CYP within 48 h following admission to intensive care for septic illness (n = 18), meningoencephalitis (n = 19) and other critical illnesses (n = 34). No associations were found between inflammatory markers and self-reported PTSS measured at 3-6 months post-discharge, or between abnormal biomarker levels and probable PTSD diagnosis.

The aforementioned study *by Pervanidou* et al. (CYP hospitalised post-RTA, n = 60; healthy controls, n = 40) found morning serum interleukin-6 at one-month post-accident to be positively associated with PTSD diagnosis (n = 9) six-months later, but not concurrently (n = 23) [35].

These two small and heterogeneous studies do not provide consistent evidence that peripheral inflammatory markers prospectively predict PTSD in CYP. Given the very small number of studies and differences in biomarkers measured, timing of sampling, and clinical context, the current evidence is insufficient to draw conclusions about inflammatory pathways in the development of PTSD.

## Cardiac

Seven studies examined posttrauma resting HR as a predictor of later PTSD outcomes, all in injured CYP presenting to hospitals. Two studies examined reactivity in cardiac parameters among maltreated or injured CYP.

### Post-injury resting heart rate (HR)

Across four studies of injured CYP presenting to hospital emergency departments (samples ranging from n = 62 to n = 190), resting HR within 24 h of presentation was found to be a positive predictor of PTSD diagnostic status at 6-months [41, 42], PTSS at 6-months [43] or PTSS at 12-months posttrauma [44], albeit with some variation in how HR was captured across studies. In the largest of these studies, *Kassam-Adams* et al. reported that elevated ED HR (defined as >2 SDs above the mean for age/gender) doubled the odds of partial or full PTSD at 6-month follow-up. Despite this consistency, there were also some mixed findings. *Olsson* et al. reported that among injured CYP aged 7-16 years (n = 79), HR upon ED admission, but not at 24 h post-admission, was higher among CYP with PTSD/subsyndromal PTSD at 6-months post-injury (n = 7) compared to those without [45]. No equivalent associations were found at 1-month post-injury. *Nixon* et al. reported that mean HR among injured CYP at triage (n = 48) [46] was predictive of self-reported PTSS at four weeks, but not at six months post-injury*. Nugent* et al. examined records-based HR during transportation by emergency services, upon hospital admission, for the first 20 min following admission, and upon discharge among 82 injured CYP [47]. HR during transportation and over the first 20 min following admission predicted 6-week PTSS measured by clinical interview. *Marsac* et al. found no associations between mean ED HR and self-reported PTSS at 2 weeks, 6 weeks, or 12 weeks post-injury among injured CYP (n = 96) [48].

**Fig. 3:**
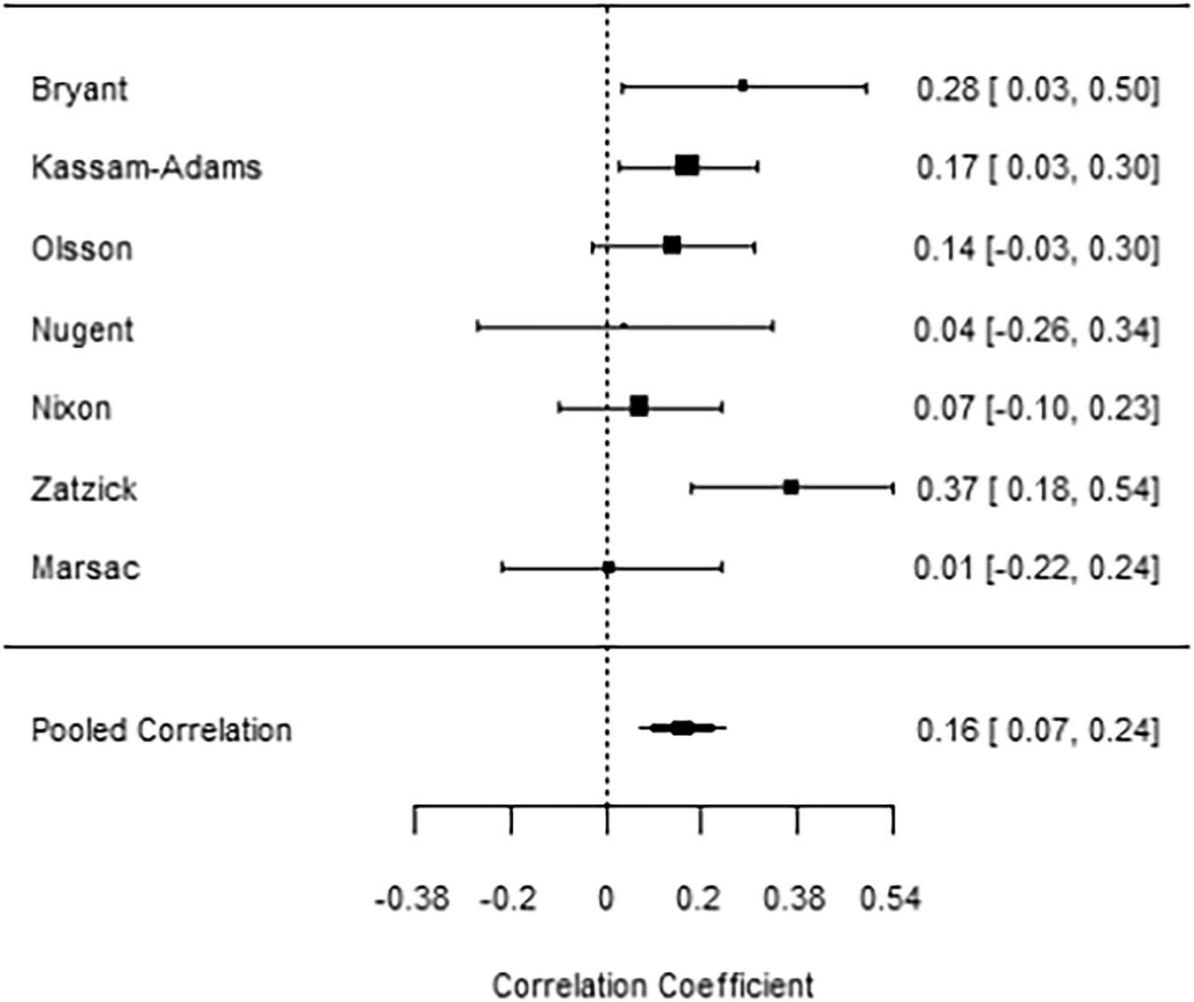
Forest Plot depicting individual and the weighted effect sizes from studies assessing the association of HR in the acute phase posttrauma with PTSD outcomes. Derived heterogeneity statistics were I² = 28.59%, and Q = 8.86, df = 6, p = 0.18. A correlation coefficient >0 indicates a positive association between HR and PTSD symptoms, a correlation coefficient <0 indicates a negative association between HR and PTSD symptoms.

**Fig. 4:**
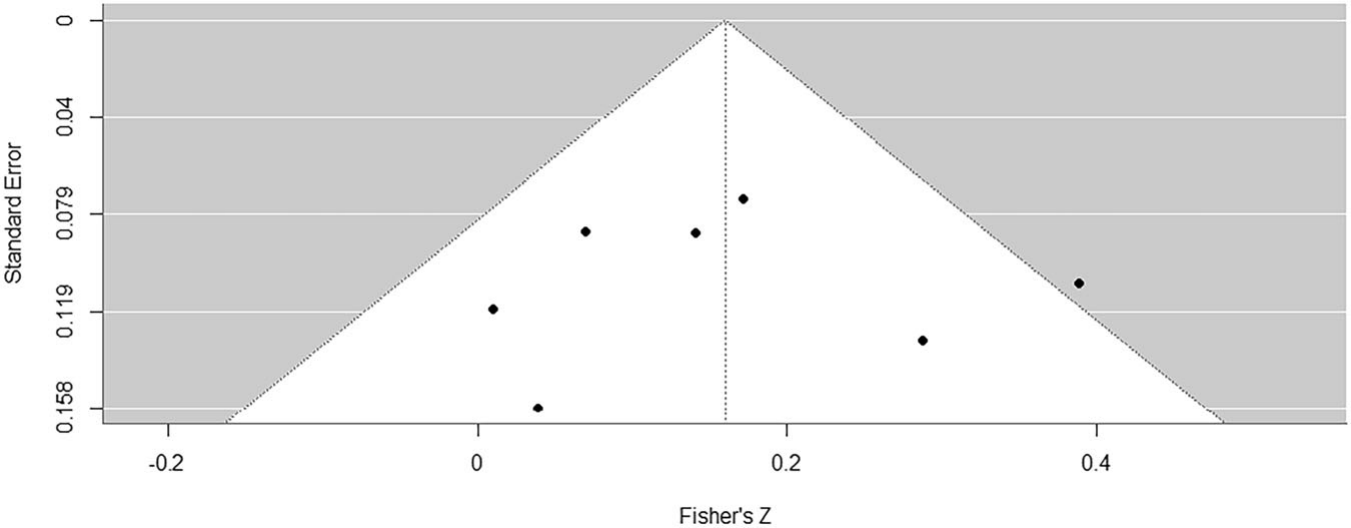
Funnel Plot depicting effect sizes and standard errors from studies assessing the association of HR in the acute phase posttrauma with PTSD outcomes.

Random-effects meta-analysis confirmed this relatively consistent pattern of predictive effects in relation to acute-phase HR, with a pooled Fisher’s Z estimate of 0.16 (*95% CI*: 0.07 to 0.25), indicating a robust positive correlation between HR in the acute phase post-trauma and later PTSD outcome (back-transformed pooled correlation coefficient = 0.16) (Fig. 3). Heterogeneity statistics were *I²* = 28.59%, and *Q* = 8.86, *df* = 6, *p* = 0.18, suggesting low-to-moderate heterogeneity that does not substantially impact the overall pooled effect size. The derived funnel plot showed a slight asymmetry around the pooled correlation estimate, with a few more studies displaying positive correlations (Fig. 4). Although this is not pronounced enough to indicate publication bias, the small number of studies limits the interpretative power of the funnel plot. Consequently, additional studies would be needed to confirm the absence of bias. Future analyses should apply pre-registered quantitative approaches such as trial sequential analysis to formally evaluate the stability of the pooled HR–PTSD association, and assess whether further data are likely to change this finding [49, 50].

### HR reactivity

Two studies examined reactivity in HR parameters. *Shenk* et al. examined respiratory sinus arrhythmia (RSA), a measure of the parasympathetic nervous system activity, during a stressor task among female adolescents who had experienced maltreatment (n = 51) and an unexposed comparison group (n = 59). The authors found no evidence that RSA at baseline was predictive of PTSS 1-year later. Haag et al. [51] examined changes in HR parameters during trauma recall relative to baseline among 76 injured CYP. Higher HR reactivity at 1-month post-trauma was positively associated with PTSS cross-sectionally and at 3-months follow-up, but not at 6-month follow-up. Reactivity in HR variability showed inverse associations with PTSS at all three timepoints, but longitudinal effects attenuated to the null when adjusting for baseline symptoms.

In summary, across emergency department cohorts of injured CYP, higher resting HR in the acute post-trauma period shows a small but relatively consistent association with later PTSD/PTSS, with a pooled correlation of around r = 0.16 and modest between-study heterogeneity. Findings for heart rate reactivity beyond the immediate post-injury phase are mixed, with some short-term associations that do not persist over longer follow-up or after accounting for baseline symptoms.

## Risk of bias assessment

Risk of bias scores ranged from 4 – 7 (maximum possible 8), capturing cohort representativeness, reliability of PTSD and biomarker assessments, adequacy of follow-up, and control of baseline PTSD symptoms and other confounding factors. Higher scores were observed for studies assessing cardiac biomarkers (Supplementary Table 2). Across studies, PTSD symptoms/diagnosis and biological or physiological markers were consistently measured using validated methods or protocols (Table 1). The follow-up period in all studies met the criterion of a minimum of four weeks, providing sufficient time to observe PTSD symptom progression. Follow-up retention was generally reported as high, with most studies retaining over 80% of participants, minimizing attrition bias. However, only a minority of studies controlled for baseline PTSD symptoms in analyses [32, 44, 48, 51]. In addition, only 14 studies included a minimum set of covariates in their analyses, controlling for age and gender by design or statistically. None of the included studies controlled for an ideal set of covariates, (age, gender, ethnicity, objective trauma severity, and a measure of socioeconomic status), meaning effect estimates are likely to be biased.

## Discussion

Our systematic search identified a limited longitudinal evidence base examining biomarker prediction of PTSD in CYP, focused on autonomic activity, cortisol secretion, and inflammatory biomarkers. No studies investigating longitudinal prediction of child PTSD via gastrointestinal, metabolomic, or epigenomic markers were identified. The majority of studies focused on the naturalistic recovery of CYP exposed to acute trauma, with a minority examining PTSS/PTSD among groups experiencing chronic trauma (maltreatment), or predictors/correlates of recovery response to treatment for PTSD. Almost all evidence derived from high income countries, with only two studies presenting evidence from middle income country CYP (China and Brazil). Only a single biomarker, HR in the immediate aftermath of acute trauma, was examined by multiple, well-powered longitudinal studies. These findings highlight the striking gap in high-quality, well powered, prospective evidence for leading biological theories in the child PTSD field.

Our review highlighted relatively consistent evidence that child HR measured within hours of presenting to hospital following trauma exposure was a positive predictor of PTSD six- to 12-months later, albeit with some inconsistency in how HR was captured and analysed across studies. Meta-analysis generated a pooled estimate indicating a robust positive correlation between HR in the acute phase post-trauma and later PTSD outcomes in CYP, consolidating prior meta-analytic findings for this biomarker. This association is assumed to be underpinned by nor-/adrenergic transmission, which can enhance fear conditioning and/or fear memory consolidation [52–54]. This model suggests that elevated HR in the acute phase post-trauma may reflect a heightened noradrenergic response and be predictive of those at risk of PTSD development [55].Practically, HR is an attractive candidate because it is routinely measured in acute care settings and can be monitored non-invasively. Our findings suggest HR in the acute-phase of a traumatic event may serve as a useful marker to identify CYP at risk of developing persistent PTSD symptoms, and that interventions aimed at reducing ANS overactivity in acute care settings may be of benefit (e.g., stress-reducing elements of psychological first-aid, biofeedback approaches). Importantly, ANS overactivity may also influence the persistence of PTSD through mechanisms including enhanced stress-related responding, impaired fear inhibition, or altered retrieval of trauma memories [56]. To move towards clinical application, this finding needs replication in larger and more diverse samples, incorporation into multivariable risk models alongside clinical and psychosocial factors, and formal testing of HR-informed, targeted early interventions in randomised trials.

Only two longitudinal studies were found to examine underlying catecholamine activity in CYP, with one finding urinary adrenalin [29] and the other plasma norepinephrine noradrenalin [34] within 24 h of child hospital admission to predict subsequent PTSD. Both studies were limited by relatively small samples and low rates of PTSD at follow-up, and therefore require replication. Moreover, beyond this evidence of the acute response to trauma, there has been almost no study of whether ANS disturbances likely contribute to ongoing PTSD in trauma exposed CYP. Only two longitudinal studies have examined cardiac reactivity beyond the acute hospitalisation phase, yielding tentative evidence that higher reactivity/lower regulation during trauma recall was predictive of later child PTSS in CYP exposed to acute trauma [51]; and no evidence that RSA in response to a stress task was predictive of later PTSD in relation to child maltreatment [57]. Large-scale longitudinal research is needed to provide a fuller understanding of ANS disturbances in PTSD, including how activity might influence PTSD maintenance or recovery beyond the immediate trauma response.

Despite biological models proposing that fundamental alterations in HPA axis activity are central to the disturbed stress responding that characterises PTSD, our review highlighted this has been very poorly studied in CYP. Although cortisol was a frequently studied biomarker (N = 10), studies were highly heterogeneous in terms of sample type (hair, urine, saliva, serum), timing, context of sampling, and analytic approach. These differences likely contribute to the inconsistent pattern of findings observed and precluded quantitative synthesis. While some studies identified longitudinal, positive associations between cortisol and PTSD, these effects tended to be limited to certain assessment points or to a subset of the study population.

The majority of studies had methodological limitations pertaining to how cortisol was measured or to the study sample, with only one of the 12 studies identified in this area having a sample of greater than 60 trauma exposed CYP. A key methodological consideration in the measurement of systemic cortisol is the impact of diurnal variation [58]. Cortisol production follows a well-established circadian rhythm, with peak levels typically occurring shortly after waking and declining throughout the day. Failure to control for time of sample collection can therefore confound associations with clinical outcomes. Among the studies included in this review, a subset employed appropriate controls for time of day. *Usta* et al. standardized blood sample collection to a 2-hour morning window following overnight fasting [37], whilst in the study sample described in *Pervanidou* et al. salivary cortisol was collected at standardised time points throughout the day, and blood samples obtained during morning fasting conditions [27, 31, 32]. In contrast, some studies measured biomarkers over extended periods (e.g., 12- or 15-hour urine samples) without clearly anchoring to consistent start times, limiting interpretability of results [29, 33]. Our review highlights the importance of methodological control when testing the association of temporally-sensitive biomarkers with PTSD outcomes, which will help improve reproducibility. In addition, studies that tracked cortisol secretion over time in relation to PTSD were lacking, despite disorder staging hypotheses in the literature which suggest cortisol hypersecretion in the short term may revert to hyposecretion in cases of prolonged PTSD [9]. Only two studies looked at cortisol stress-reactivity, both focusing on CYP exposed to maltreatment, despite the clear relevance of understanding the role of stress responding in the context of trauma and PTSD. Beyond these methodological limitations, discrepancies from the adult PTSD literature are also notable: meta-analytic and experimental work has linked PTSD in adults to modest alterations in HPA axis function, including lower basal cortisol in many chronic PTSD samples[23]. It is plausible that the absence of a clear, uniform cortisol signal in CYP reflects age-dependent neurobiological trajectories and differences in trauma history and type, rather than a lack of HPA involvement in aetiology.

Cross-sectional studies of adult PTSD have found pronounced immune differences, including higher concentrations of inflammatory biomarkers [13, 59]. This is consistent with observations that individuals with PTSD have a higher burden of inflammatory disease, including increased rates of cardiovascular disease, diabetes mellitus, and autoimmune diseases, and have premature mortality [60]. Nevertheless, while peripheral immune activation appears to be associated with PTSD, a causal direction of these associations has not been proven [59]. Recent evidence from a study using Mendelian Randomization, a genetically informed study design more robust to confounding, suggested a bidirectional causal relationship between PTSD and CRP [61]. It is possible that systemic inflammation following trauma could lead to psychopathology by altering metabolism or availability of key neurotransmitters, or by influencing the function of the brain-resident immune cells triggering neuroinflammatory cascades. Conversely, inflammation may develop as a consequence of high levels of arousal in PTSD, presumably linked to hyperactivation of the ANS and impairment in LHPA axis functioning [13, 62, 63]. Longitudinal studies in childhood may be particularly important to disentangling these possibilities, given that many non-communicable diseases and health risk behaviours associated with inflammation have yet to emerge. However, only two small studies in our review examined inflammatory markers in relation to later child PTSD, with inconsistent findings. Further, robust evidence on inflammatory biomarkers in relation to PTSD in CYP would allow insight into the long-term alterations identified in adults and associated adverse health outcomes.

A key limitation in the identified evidence base is the small sample size of individual studies and the lack of replication using standardised methods. Only a single biomarker, HR in the acute phase post-trauma, with positive associations replicated across multiple well-powered studies, was identified as a promising prospective biomarker of youth PTSD. In contrast, whilst cortisol was frequently studied, our narrative synthesis found no consistent association between cortisol levels and PTSD outcomes in CYP. However, this finding should not be interpreted as evidence of a true null relationship, which would need to be confirmed in multiple, adequately powered samples using harmonised methodologies. A lack of statistical power increases the risk of both Type II (false negatives) and Type I error (false positives), which is likely to have contributed to the contradictory findings. It is also important to note this synthesis was restricted to peer-reviewed literature, and therefore our findings may be impacted by publication bias [64].

Taken together, the current literature does not support strong conclusions regarding the biomarker candidates assessed, though preliminary support for HR in the acute phase post-trauma as an early risk indicator provides a potentially valuable direction for future research. Well-powered longitudinal studies, using harmonised methodologies, are warranted to determine whether prospective biomarkers identified in adult PTSD populations translate meaningfully to younger cohorts.

## Conclusions

Longitudinal studies present a major opportunity to identify causal factors in disorder aetiology and are particularly important in the PTSD field, where there is a 6-month window posttrauma during which early symptoms typically either resolve or become chronic [3, 65]. Understanding the biological factors that underpin differential posttrauma recovery trajectories in this key window is particularly critical to 1) identifying CYP who are vulnerable to developing PTSD posttrauma, and 2) informing novel and/or targeted intervention development. Nonetheless, our systematic review for prospective biomarkers of childhood PTSD development and persistence revealed a limited evidence base, with only HR posttrauma yielding consistent predictive effects. It is important to note this should be viewed as an emerging biomarker for PTSD risk, and will require further validation in diverse trauma contexts and in combination with other clinical and psychosocial risk factors. Hormonal and inflammatory markers were subject to more limited study, and prospective evidence for metabolomic, gastrointestinal, and epigenomic markers of risk for PTSD was absent, despite studies in adult sample suggesting their relevance. Understanding the psychobiological processes that underpin childhood PTSD remains a major research goal that will allow for the development of novel therapeutic strategies. Further high-quality longitudinal research, including multi-level integration of biological and psychosocial determinants of risk for and persistence of PTSD in CYP, is critical to advancing the field.

## Supplementary information

Supplementary materials
