# Supplementary material for: Prospective biomarkers of posttraumatic stress disorder in children and adolescents: a systematic review and meta-analysis
Source: Transl Psychiatry. 2026 Mar 30;16:217. doi: 10.1038/s41398-026-03939-1 (PMC13039113; doi:10.1038/s41398-026-03939-1)
Supplement: Supplementary file 1 — Supplementary materials [file 41398_2026_3939_MOESM1_ESM.docx]

**Supplementary Materials**

| **Section and Topic** | **Item #** | **Checklist item** |
| --- | --- | --- |
| **TITLE** | | |
| Title | 1 | Identify the report as a systematic review. |
| **ABSTRACT** | | |
| Abstract | 2 | See the PRISMA 2020 for Abstracts checklist. |
| **INTRODUCTION** | | |
| Rationale | 3 | Describe the rationale for the review in the context of existing knowledge. |
| Objectives | 4 | Provide an explicit statement of the objective(s) or question(s) the review addresses. |
| **METHODS** | | |
| Eligibility criteria | 5 | Specify the inclusion and exclusion criteria for the review and how studies were grouped for the syntheses. |
| Information sources | 6 | Specify all databases, registers, websites, organisations, reference lists and other sources searched or consulted to identify studies. Specify the date when each source was last searched or consulted. |
| Search strategy | 7 | Present the full search strategies for all databases, registers and websites, including any filters and limits used. |
| Selection process | 8 | Specify the methods used to decide whether a study met the inclusion criteria of the review, including how many reviewers screened each record and each report retrieved, whether they worked independently, and if applicable, details of automation tools used in the process. |
| Data collection process | 9 | Specify the methods used to collect data from reports, including how many reviewers collected data from each report, whether they worked independently, any processes for obtaining or confirming data from study investigators, and if applicable, details of automation tools used in the process. |
| Data items | 10a | List and define all outcomes for which data were sought. Specify whether all results that were compatible with each outcome domain in each study were sought (e.g., for all measures, time points, analyses), and if not, the methods used to decide which results to collect. |
|  | 10b | List and define all other variables for which data were sought (e.g., participant and intervention characteristics, funding sources). Describe any assumptions made about any missing or unclear information. |
| Study risk of bias assessment | 11 | Specify the methods used to assess risk of bias in the included studies, including details of the tool(s) used, how many reviewers assessed each study and whether they worked independently, and if applicable, details of automation tools used in the process. |
| Effect measures | 12 | Specify for each outcome the effect measure(s) (e.g., risk ratio, mean difference) used in the synthesis or presentation of results. |
| Synthesis methods | 13a | Describe the processes used to decide which studies were eligible for each synthesis (e.g., tabulating the study intervention characteristics and comparing against the planned groups for each synthesis (item #5)). |
|  | 13b | Describe any methods required to prepare the data for presentation or synthesis, such as handling of missing summary statistics, or data conversions. |
|  | 13c | Describe any methods used to tabulate or visually display results of individual studies and syntheses. |
|  | 13d | Describe any methods used to synthesize results and provide a rationale for the choice(s). If meta-analysis was performed, describe the model(s), method(s) to identify the presence and extent of statistical heterogeneity, and software package(s) used. |
|  | 13e | Describe any methods used to explore possible causes of heterogeneity among study results (e.g., subgroup analysis, meta-regression). |
|  | 13f | Describe any sensitivity analyses conducted to assess robustness of the synthesized results. |
| Reporting bias assessment | 14 | Describe any methods used to assess risk of bias due to missing results in a synthesis (arising from reporting biases). |
| Certainty assessment | 15 | Describe any methods used to assess certainty (or confidence) in the body of evidence for an outcome. |
| **RESULTS** | | |
| Study selection | 16a | Describe the results of the search and selection process, from the number of records identified in the search to the number of studies included in the review, ideally using a flow diagram. |
|  | 16b | Cite studies that might appear to meet the inclusion criteria, but which were excluded, and explain why they were excluded. |
| Study characteristics | 17 | Cite each included study and present its characteristics. |
| Risk of bias in studies | 18 | Present assessments of risk of bias for each included study. |
| Results of individual studies | 19 | For all outcomes, present, for each study: (a) summary statistics for each group (where appropriate) and (b) an effect estimate and its precision (e.g., confidence/credible interval), ideally using structured tables or plots. |
| Results of syntheses | 20a | For each synthesis, briefly summarise the characteristics and risk of bias among contributing studies. |
|  | 20b | Present results of all statistical syntheses conducted. If meta-analysis was done, present for each the summary estimate and its precision (e.g., confidence/credible interval) and measures of statistical heterogeneity. If comparing groups, describe the direction of the effect. |
|  | 20c | Present results of all investigations of possible causes of heterogeneity among study results. |
|  | 20d | Present results of all sensitivity analyses conducted to assess the robustness of the synthesized results. |
| Reporting biases | 21 | Present assessments of risk of bias due to missing results (arising from reporting biases) for each synthesis assessed. |
| Certainty of evidence | 22 | Present assessments of certainty (or confidence) in the body of evidence for each outcome assessed. |
| **DISCUSSION** | | |
| Discussion | 23a | Provide a general interpretation of the results in the context of other evidence. |
|  | 23b | Discuss any limitations of the evidence included in the review. |
|  | 23c | Discuss any limitations of the review processes used. |
|  | 23d | Discuss implications of the results for practice, policy, and future research. |
| **OTHER INFORMATION** | | |
| Registration and protocol | 24a | Provide registration information for the review, including register name and registration number, or state that the review was not registered. |
|  | 24b | Indicate where the review protocol can be accessed, or state that a protocol was not prepared. |
|  | 24c | Describe and explain any amendments to information provided at registration or in the protocol. |
| Support | 25 | Describe sources of financial or non-financial support for the review, and the role of the funders or sponsors in the review. |
| Competing interests | 26 | Declare any competing interests of review authors. |
| Availability of data, code and other materials | 27 | Report which of the following are publicly available and where they can be found: template data collection forms; data extracted from included studies; data used for all analyses; analytic code; any other materials used in the review. |

Table S1: PRISMA 2020 checklist.

**Supplementary Methods**

**Search Strategy**

1. (PTSD or post-trauma* or PTSS or posttrauma*).ti, ab.

2. (child* or infan* or teen* or adoles* or youth or preschool*).ti, ab.

3. (serotonin or dopamin* or norepinephrine or noradrenalin* or glutamic or glutama* or acetylcholin* or cholinergic* or epigenome* or EWAS or methylation or hormone or steroid* or estradiol* or oestrogen or estrogen or progesterone or FSH or follicle stimulating horome or cortisol or adrenalin* or thyroxine or endorphin or glucocorticoid or cholesterol or T3 or T4 or heartrate or heart* or cardiac or catecholamine or ECG or electrocardiogram or CRP or C-reactive protein or ESR or erythrocyte or albumin or interferon or cytokine* or interleukin* or microbiota or microbiome or fecal or gut-brain or gut or MRI or fMRI or magnetic resonance or imaging or EEG or electroencephalography or electroencephalogram or DTI or diffusion tensor imaging or brain imaging or MEG or magnetoencephalography or PET or Positron emission tomography or biomarker or biological marker or clinical marker or serum marker or metabolic marker or metabolo* or proteom* or body mass index or BMI or blood glucose).ti, ab.

4. [1] AND [2] AND [3]

Eligibility criteria

We screened for studies assessing associations between biological or physiological markers and a later PTSD outcome in CYP using the following inclusion criteria: English language peer-reviewed publication; human participants with a mean age 18-years old or younger; biomarker exposure captured prospectively (i.e., before PTSD/PTSS was assessed); valid PTSD/PTSS outcome measure (dimensional or categorical); data captured at least two timepoints; study design to include observational, cohort studies, randomised-controlled trials and quasi-randomised controlled trials. Studies using a pseudo-longitudinal design, such as those measuring biological markers in hair samples (which capture cumulative data over time as hair grows), were eligible.

Assessment of Risk of Bias

The adapted Newcastle Ottawa Scale (NOS) focussed on six domains: (1) cohort selection, (2) quality and validity of the PTSD assessment instrument, (3) adjustment for baseline PTSD symptoms, (4) adjustment for covariates (by matching, stratification, or statistical adjustment) (5) quality and reporting of biomarker assessment, (6) adequacy of follow-up.

We defined minimum (age and gender) and ideal (age, gender, ethnicity, objective trauma severity where applicable (e.g., triage category) and socioeconomic status) covariate sets *a priori*, based on expert consensus that these are robust predictors of PTSD risk.

It is important to note that both sex- (the set of biological attributes that are associated with physical and physiological features) and gender- (*t*he socially constructed roles, behaviours and identities of female, male and gender-diverse people ) based differences in PTSD are well-documented (1, 2). This distinction is particularly relevant when considering psychobiological aetiology. The importance of systematically integrating sex and gender in PTSD research has previously been described, which includes the use of validated questionnaires to capture these measures separately, and accurate reporting (3). When discussing the evidence base, we therefore use the more inclusive term of gender, but note that this distinction was generally not made clear in included studies.

Meta-analysis

Univariate product-moment correlation coefficients were used as effect sizes, following methods described previously (4). Correlation coefficients were first transformed into Fisher's Z values to stabilize variance, and effect sizes and weights were combined into a weighted mean effect size with corresponding 95% confidence intervals. For ease of interpretation, effect sizes were then back-transformed to a product-moment correlation coefficient. A random-effects model was employed to account for potential heterogeneity among the studies. The pooled Fisher's Z estimate and its corresponding 95% confidence intervals (CIs) were calculated. The degree of heterogeneity was assessed using the I² statistic and the Q-test. Publication bias was explored through the visual inspection of funnel plots.

|  |  | 1. Cohort representative of target population and control group recruited from same population?^1^ | 2. Can we be confident in the assessment of PTSD?^2^ | 3. Can we be confident in the assessment of the biological or physiological marker>? | 4. Were baseline PTSD symptoms controlled for? | 5. Were a minimum set of confounding variables controlled for (by design or statistically)?^3^ | 6. Were an ideal set of confounding variables controlled for (by design or statistically)?^4^ | 7. Was the follow-up period sufficient? ^5^ | 8. Was the follow-up of cohorts adequate?^6^ | Quality score (Total number) |
| --- | --- | --- | --- | --- | --- | --- | --- | --- | --- | --- |
| Bryant et al. (2007) | Cardiac | ✔ | ✔ | ✔ | **?** | ✔ | **X** | ✔ | ✔ | **6** |
| Deyoung et al. (2007) | Cardiac | ✔ | ✔ | ✔ | **?** | ✔ | **X** | ✔ | ✔ | **5** |
| Haag et al. (2009) | Cardiac | ✔ | ✔ | ✔ | ✔ | ✔ | **X** | ✔ | ✔ | **7** |
| Kassam-Adams et al. (2005) | Cardiac | ✔ | ✔ | ✔ | **X** | ✔ | **X** | ✔ | ✔ | **6** |
| Marsac et al. (2017) | Cardiac | ✔ | ✔ | ✔ | ✔ | ✔ | **X** | ✔ | **X** | **6** |
| Nixon et al. (2010) | Cardiac | ✔ | ✔ | ✔ | **?** | **X** | **X** | ✔ | ✔ | **5** |
| Nugent et al. (2006) | Cardiac | ✔ | ✔ | ✔ | **X** | **X** | **X** | ✔ | **X** | **4** |
| Olsson et al. (2008) | Cardiac | ✔ | ✔ | ✔ | **?** | **X** | **X** | ✔ | **?** | **4** |
| Zatzick et al. (2006) | Cardiac | ✔ | ✔ | ✔ | ✔ | ✔ | **X** | ✔ | ✔ | **7** |
| Nugent et al. (2006) | Cardiac and hormonal | ✔ | ✔ | ✔ | **X** | **X** | **X** | ✔ | **X** | **4** |
| Shenk et al. (2014) | Cardiac and hormonal | **X** | ✔ | ✔ | **X** | ✔ | **X** | ✔ | ✔ | **5** |
| Boeckel et al. (2017) | Hormonal | ✔ | ✔ | ✔ | **NA*** | ✔ | **X** | ✔ | ✔ | **7** |
| Caspani et al. (2018) | Hormonal | ✔ | ✔ | ✔ | **X** | ✔ | **X** | ✔ | ✔ | **6** |
| Delhanty et al. (2005) | Hormonal | ✔ | ✔ | ✔ | **X** | ✔ | **X** | ✔ | ✔ | **6** |
| Kolatis et al. (2011) | Hormonal | ✔ | ✔ | ✔ | **X** | ✔ | **X** | ✔ | ✔ | **6** |
| Luo et al., (2012) | Hormonal | ✔ | ✔ | ✔ | **X** | ✔ | **X** | ✔ | ✔ | **5** |
| Negriff et al. (2021) | Hormonal | **X** | ✔ | ✔ | ✔ | ✔ | **X** | ✔ | ✔ | **6** |
| Ostrowski et al. (2007) | Hormonal | ✔ | ✔ | ✔ | **X** | **X** | **X** | ✔ | **X** | **4** |
| Pervanidou et al. (2007a) | Hormonal | ✔ | ✔ | ✔ | **X** | **X** | **X** | ✔ | ✔ | **5** |
| Straub et al. (2017) | Hormonal | ✔ | ✔ | ✔ | **X** | **X** | **X** | ✔ | ✔ | **5** |
| Usta et al. (2018) | Hormonal | ✔ | ✔ | ✔ | **X** | ✔ | **X** | ✔ | ✔ | **6** |
| Pervanidou et al. (2007b) | Hormonal and immunological | ✔ | ✔ | ✔ | **X** | **X** | **X** | ✔ | ✔ | **5** |

Table S2: Risk of bias assessment for included studies. ^1^Five articles included a control group unexposed to trauma in their study design (5-9). ^2^Based on reported use of a validated tool for assessment of PTSD.^3^A minimum set of confounding variables required to adequately control for bias were defined as: age and sex. ^4^An ideal set of confounding variables required to adequately control for bias were defined as: age, sex, ethnicity, objective trauma severity (where applicable), and a measure of socio-economic status.^5^An adequate follow-period defined as more than 4 weeks. ^6^An adequate follow-up rate defined as >80%.*Not applicable due to participant age.

| **Study ID** | **Biomarker** | **Analysis** | **Comparisons/Timepoints** | **Effect size** | **p-value** |  |  |  |
| --- | --- | --- | --- | --- | --- | --- | --- | --- |
| **Luo et al., 2012** |  |  |  |  |  |  |  |  |
|  |  | Chi-squared | PSTD at 1 month vs. non-PTSD | F = 3.88 | 0.0499 |  |  |  |
|  |  |  | PSTD at 1 month vs. control | F = 6.27 | 0.0130 |  |  |  |
|  |  |  | PTSD 2-4 months vs non-PTSD | F = 6.17 | 0.0137 |  |  |  |
|  |  |  | non-PTSD AT 2-4 months vs. control | F = 11.74 | 0.0007 |  |  |  |
|  |  |  | PTSD 5-7 months vs non-PTSD | F = 4.11 | 0.0438 |  |  |  |
|  |  | MLM | Overall | F = 3.27 | 0.0042 |  |  |  |
| **Kolaitis et al., 2011** |  |  |  | **Effect size** | **95% CI** | **p-value** |  |  |
|  | Cortisol | Logistic regression | 6-months PTSD vs. no PTSD | OR = 1.006 | 1.001 - 1.011 | <.05 |  |  |
| **Pervanidou et al., 2007a** |  |  |  | **Group effect** |  | **p-value** |  |  |
|  | Cortisol | 3 x 3 repeated measures ANOVA | Repeated | F(2,54) = 5.885 | 0.179 | 0.005 |  |  |
|  | Noradrenaline |  |  | F(2,62) = 8.859 | 0.222 | <.001 |  |  |
|  | Cortisol |  | 1-month PTSD vs. non-PTSD | *Not reported* | *Not reported* | >.005 |  |  |
|  | Noradrenaline |  |  | *Not reported* | *Not reported* | 0.02 |  |  |
|  | Cortisol |  | 6-month PTSD vs. non-PTSD | *Not reported* | *Not reported* | >.005 |  |  |
|  | Noradrenaline |  |  | *Not reported* | *Not reported* | 0.006 |  |  |
| **Pervanidou et al., 2007b** |  |  |  | **Beta** | **t** | **p-value** |  |  |
|  | Cortisol | Hierarchical regression | 1-month PTSD vs. non-PTSD | *Not reported* | *Not reported* | >.05 |  |  |
|  | IL-6 |  |  | *Not reported* | *Not reported* | >.05 |  |  |
|  | Cortisol |  | 6-month PTSD vs. non-PTSD | 0.463 | 3.038 | 0.004 |  |  |
|  | IL-6 |  |  | 0.450 | 3.040 | 0.004 |  |  |
| **Straub et al., 2017** |  |  |  | **r** | **p-value** |  |  |  |
|  | Cortisol | Spearman correlations | 3-month PTSS score | -0.13 | 0.47 |  |  |  |
|  |  |  | 3-month PTSS score (sex stratified - girls) | -0.56 | 0.06 |  |  |  |
|  |  |  | 3-month PTSS score (sex stratified - boys) | 0.07 | 0.74 |  |  |  |
| **Boeckel et al., 2017** |  |  |  | **r** | **p-value** |  |  |  |
|  | Cortisol | Spearman correlations | Baseline PTSS score/IPV exposed | *Not reported* | >.05 |  |  |  |
|  |  |  | Baseline PTSS score/controls | *Not reported* | >.05 |  |  |  |
| **Usta et al., 2018** |  |  |  | **AUC** | **95% CI** | **p-value** |  |  |
|  | DHEA-S | ROC analysis | Positive response vs non-response | 0.611 | 0.420-0.802 | 0.234 |  |  |
|  | Cortisol |  | Positive response vs non-response | 0.391 | 0.214-0.569 | 0.245 |  |  |
|  | DHEA/Cortisol |  | Positive response vs non-response | 0.703 | 0.530-0.877 | 0.03 |  |  |
| **Ostrowski et al., 2007** |  |  |  | **B** | **SE B** | **Coefficient** | **R2** | **p-value** |
|  | Cortisol | Hierarchical linear regression | 6-week PTSS (total sample) | *Not reported* | *Not reported* | *Not reported* | *Not reported* | >.05 |
|  |  |  | 6-week PTSS (-prior trauma) | 0.01 | 0 | 0.36 | 0.12 | <.05 |
|  |  |  | 6-week PTSS (-prior trauma) | *Not reported* | *Not reported* | *Not reported* | 0.09 | 0.07 |
|  |  |  | 7-month PTSS (total sample) | *Not reported* | *Not reported* | *Not reported* | *Not reported* | >.05 |
|  |  |  | 7-month PTSS (-prior trauma) | *Not reported* | *Not reported* | *Not reported* | *Not reported* | >.20 |
|  |  |  | 7-month PTSS (-prior trauma, boys) | *Not reported* | *Not reported* | *Not reported* | 0.23 | <.05 |
|  |  |  | 7-month PTSS (-prior trauma, girls) | *Not reported* | *Not reported* | *Not reported* | 0.01 | >.05 |
| **Delahanty et al., 2005** |  |  |  | **R2** | **p-value** | **Coefficient** | **Delta R2** |  |
|  | Cortisol | Hierarchical linear regression | 6-week PTSS | 0.413 | <.001 | 0.329 | 0.103 |  |
|  | Epinephrine |  | 6-week PTSS | 0.387 | <.001 | 0.277 | 0.065 |  |
|  | Norepinephrine |  | 6-week PTSS | *Not reported* | >.05 | *Not reported* | *Not reported* |  |
|  | Dopamine |  | 6-week PTSS | *Not reported* | >.05 | *Not reported* | *Not reported* |  |
| **Negriff et al., 2021** |  |  |  | **Coefficient** | **p-value** |  |  |  |
|  | Cortisol | Cross-lagged multiple-group path models | T1 AUCg on T2 PTSS in SA group | -0.03 | <.01 |  |  |  |
|  |  |  | T1 AUCg on T2 PTSS in PA group | 0.01 | >.05 |  |  |  |
|  |  |  | T1 AUCg on T2 PTSS in EA group | 0.16 | >.05 |  |  |  |
|  |  |  | T1 AUCg on T2 PTSS in Neg group | 0.39 | >.05 |  |  |  |
|  |  |  | T1 AUCg on T2 PTSS in control group | -0.02 | >.05 |  |  |  |

Table S3: Results extracted from identified studies assessing association of a prospective hormonal biomarker measure with a PTSD outcome. PTSD (posttraumatic stress disorder), PTSS ()IL-6 (Interleukin 6), DHEA (Dehydroepiandrosterone), MLM (multilevel modelling).

| **Study ID** | **Biomarker** | **Analysis** | **Comparison/timepoint** |  |  |  |
| --- | --- | --- | --- | --- | --- | --- |
|  |  |  |  | **Coefficient** | **p-value** |  |
| **Caspani et al., 2018** | White cell count | Pearson partial correlation | Full cohort | 0.171 | *not reported* |  |
|  |  |  | Meningoencephalitis | −0.037 | *not reported* |  |
|  |  |  | Septic illness | 0.16 | *not reported* |  |
|  |  |  | Other illness | 0.24 | *not reported* |  |
|  | Neutrophils |  | Full cohort | 0.15 | *not reported* |  |
|  |  |  | Meningoencephalitis | −0.085 | *not reported* |  |
|  |  |  | Septic illness | 0.16 | *not reported* |  |
|  |  |  | Other illness | 0.30 | *not reported* |  |
|  | Lymphocytes |  | Full cohort | 0.16 | *not reported* |  |
|  |  |  | Meningoencephalitis | 0.65 | *not reported* |  |
|  |  |  | Septic illness | 0.09 | *not reported* |  |
|  |  |  | Other illness | −0.065 | *not reported* |  |
|  | Platelets |  | Full cohort | −0.301 | *not reported* |  |
|  |  |  | Meningoencephalitis | −0.108 | *not reported* |  |
|  |  |  | Septic illness | −0.323 | *not reported* |  |
|  |  |  | Other illness | −0.17 | *not reported* |  |
|  | Fibrinogen |  | Full cohort | 0.20 | *not reported* |  |
|  |  |  | Meningoencephalitis | 0.18 | *not reported* |  |
|  |  |  | Septic illness | −0.418 | *not reported* |  |
|  |  |  | Other illness | −0.183 | *not reported* |  |
|  | CRP |  | Full cohort | 0.19 | *not reported* |  |
|  |  |  | Meningoencephalitis | −0.557 | *not reported* |  |
|  |  |  | Septic illness | 0.82 | <0.05 |  |
|  |  |  | Other illness | −0.204 | *not reported* |  |
|  | Lactate |  | Full cohort | −0.023 | *not reported* |  |
|  |  |  | Meningoencephalitis | −0.549 | *not reported* |  |
|  |  |  | Septic illness | −0.379 | *not reported* |  |
|  |  |  | Other illness | −0.488 | not reported |  |
| **Pervanidou et al., 2007** |  |  |  | **Beta** | **t** | **p-value** |
|  | Cortisol | Hierarchical regression | 1-month PTSD vs. non-PTSD | *Not reported* | *Not reported* | >.05 |
|  | IL-6 |  | 1-month PTSD vs. non-PTSD | *Not reported* | *Not reported* | >.05 |
|  | Cortisol |  | 6-month PTSD vs. non-PTSD | 0.463 | 3.038 | 0.004 |
|  | IL-6 |  | 6-month PTSD vs. non-PTSD | 0.450 | 3.040 | 0.004 |

Table S4: Results extracted from identified studies assessing association of a prospective immunological or immunological and hormonal biomarker measure with a PTSD outcome. PTSD (posttraumatic stress disorder), PTSS ()IL-6 (Interleukin 6).

| **Study ID** | **Biomarker** | **Analysis** | **Comparison/timepoint** |  |  |  |  |  |
| --- | --- | --- | --- | --- | --- | --- | --- | --- |
| **Zatzick et al. (2006)** |  | Random coefficient regression |  | **B** | **SE** | **p-value** |  |  |
|  | HR |  | PTSS at 1 year | 0.11 | 0.04 | 0.002 |  |  |
|  |  |  |  | **B** | **SE** | **p-value** |  |  |
| **Haag et al. (2019)** |  |  |  |  |  |  |  |  |
| *Child narrative task* | (mean) HR | linear regression | PTSS at 1-month | 0.47 | 0.21 | <0.05 |  |  |
|  | HRV: HFBP |  | PTSS at 1-month | -0.51 | 0.2 | <0.05 |  |  |
|  | HRV:LFBP |  | PTSS at 1-month | -0.32 | 0.19 | >0.05 |  |  |
|  |  |  |  |  |  |  |  |  |
|  | (mean) HR |  | PTSS at 3-months | 0.58 | 0.24 | <0.05 |  |  |
|  | HRV: HFBP |  | PTSS at 3-months | -0.24 | 0.1 | <0.05 |  |  |
|  | HRV:LFBP |  | PTSS at 3-months | -0.53 | 0.23 | <0.05 |  |  |
|  |  |  |  |  |  |  |  |  |
|  | (mean) HR |  | PTSS at 6-months | 0.36 | 0.13 | >0.05 |  |  |
|  | HRV: HFBP |  | PTSS at 6-months | -0.56 | 0.26 | <0.05 |  |  |
|  | HRV:LFBP |  | PTSS at 6-months | -0.44 | 0.25 | >0.05 |  |  |
|  |  |  |  |  |  |  |  |  |
| *Joint narrative task* | (mean) HR |  | PTSS at 1-month | 0.46 | 0.22 | <0.05 |  |  |
|  | HRV: HFBP |  | PTSS at 1-month | -0.62 | 0.21 | <0.01 |  |  |
|  | HRV:LFBP |  | PTSS at 1-month | -0.82 | 0.21 | <0.01 |  |  |
|  |  |  |  |  |  |  |  |  |
|  | (mean) HR |  | PTSS at 3-months | 0.7 | 0.22 | <0.01 |  |  |
|  | HRV: HFBP |  | PTSS at 3-months | -0.75 | 0.22 | <0.01 |  |  |
|  | HRV:LFBP |  | PTSS at 3-months | -0.65 | 0.24 | <0.05 |  |  |
|  |  |  |  |  |  |  |  |  |
|  | (mean) HR |  | PTSS at 6-months | 0.38 | 0.26 | >0.05 |  |  |
|  | HRV: HFBP |  | PTSS at 6-months | -0.67 | 0.24 | <0.01 |  |  |
|  | HRV:LFBP |  | PTSS at 6-months | -0.8 | 0.25 | <0.01 |  |  |
|  |  |  |  |  |  |  |  |  |
|  | (mean) HR |  | PTSS at 3-months | 0.46 | 0.2 | 0.003 |  |  |
| **Nugent et al. 2006** |  |  |  | **ΔR2** | **p-value** |  |  |  |
|  | HR | hierarchical linear regression | PTSS at 6 weeks | 0.068 | < .01 |  |  |  |
|  | HR (averaged) |  | PTSS at 6 weeks | 0.083 | < .01 |  |  |  |
|  | HR (averaged over 20 mins) |  | PTSS at 6 weeks | 0.047 | < .05 |  |  |  |
|  | HR |  | PTSS at 6 months | 0.06 | < .05 |  |  |  |
| **De Young et al., 2007** |  |  |  | **ΔR2** | **p-value** |  |  |  |
|  | HR | Linear regression | PTSD | 0.07 | < .05 |  |  |  |
| **Bryant et al, 2007** |  |  |  | **OR** | **95% CI** |  |  |  |
|  |  | logistic regression | full/subsyndromal PTSD at 6 months | 5.89 | 1.17–29.68 |  |  |  |
| **Kassam-Adams et al., 2005** |  |  |  | **OR** | **95% CI** |  |  |  |
|  | HR | linear regression | PTSS | 2.4 | 1.1-5.4 |  |  |  |
| **Olsson et al., 2008** |  |  |  | **Coefficient** | **p-value** |  |  |  |
|  | HR (ED) | t-test | PTSS 6 months | t(77) = 2.82 | p < .01 |  |  |  |
|  | HR (24 hours) |  | PTSS 6 months | t(77) = 3.84 | p = .079 |  |  |  |
|  | HR (ED) |  | PTSS 1 month | *not reported* | *not reported* |  |  |  |
|  | HR (24 hours) |  | PTSS 1 month | *not reported* | *not reported* |  |  |  |
| **Nixon et al., 2010** |  |  |  | **Coefficient** | **p-value** |  |  |  |
|  | HR | Inter correlation | PTSS change from 1 month to 6 month | 0.14 | > .05 |  |  |  |
| **Marsac et al., 2017** |  |  |  | **Coefficient** | **p-value** |  |  |  |
|  | HR | Bivariate analyses (correlations) | PTSS at 6-weeks | 0.18 | >0.05 |  |  |  |
|  |  |  | PTSS at 12-weeks | 0.01 | >0.05 |  |  |  |
| **Nugent et al., 2006** |  |  |  | **R2** | **Change in R2** | **p-value** | **B** | **SE** |
|  | HR | Hierarchical linear regression | PTSS at 6 weeks | 0.6 | 0.08 | <0.05 | 0.01 | 0.02 |
|  | Cortisol |  | PTSS at 6 weeks |  |  |  | 0.08 | 0.03 |
|  |  |  |  |  |  |  |  |  |
|  | HR |  | PTSS at 6 months | 0.77 | 0.18 | <0.05 | -0.01 | 0.01 |
|  | Cortisol |  | PTSS at 6 months |  |  |  | -0.05 | 0.02 |
| **Shenk et al., 2014** |  |  |  | **Coefficient** | **p-value** |  |  |  |
|  | RSA reactivity | Multiple mediator model | PTSS | -0.35 | >0.05 |  |  |  |
|  | Cortisol reactivity |  | PTSS | 0.5 | >0.05 |  |  |  |

Table S5: Results extracted from identified studies assessing association of a prospective cardiac biomarker measure with a PTSD outcome. PTSD (posttraumatic stress disorder), HR (heartrate), HRV (heart rate variability), HFBP (high frequency band power), LFBP (low frequency band power), RSA (Respiratory sinus arrhythmia).

**References**

1. Heidari S, Babor TF, De Castro P, Tort S, Curno M. Sex and Gender Equity in Research: rationale for the SAGER guidelines and recommended use. Res Integr Peer Rev. 2016;1:2.

2. Langeland W, Olff M. Sex and gender in psychotrauma research. Eur J Psychotraumatol. 2024;15(1):2358702.

3. Langevin R, Beaudette S, Wadji DL, Chabake SA, Gonzalez C, Jenkins D, et al. Sex and gender considerations in cross-cultural traumatic stress studies. Eur J Psychotraumatol. 2024;15(1):2408194.

4. Alisic E, Jongmans MJ, van Wesel F, Kleber RJ. Building child trauma theory from longitudinal studies: a meta-analysis. Clin Psychol Rev. 2011;31(5):736-47.

5. Shenk CE, Putnam FW, Rausch JR, Peugh JL, Noll JG. A longitudinal study of several potential mediators of the relationship between child maltreatment and posttraumatic stress disorder symptoms. Dev Psychopathol. 2014;26(1):81-91.

6. Boeckel MG, Viola TW, Daruy L, Martinez M, Grassi-Oliveira R. Intimate partner violence is associated with increased maternal hair cortisol in mother-child dyads. Compr Psychiat. 2017;72:18-24.

7. Pervanidou P, Kolaitis G, Charitaki S, Lazaropoulou C, Papassotiriou I, Hindmarsh P, et al. The natural history of neuroendocrine changes in pediatric posttraumatic stress disorder (PTSD) after motor vehicle accidents: progressive divergence of noradrenaline and cortisol concentrations over time. Biol Psychiatry. 2007;62(10):1095-102.

8. Negriff S, Gordis EB, Susman EJ. Associations between HPA axis reactivity and PTSD and depressive symptoms: Importance of maltreatment type and puberty. Dev Psychopathol. 2021.

9. Pervanidou P, Kolaitis G, Charitaki S, Margeli A, Ferentinos S, Bakoula C, et al. Elevated morning serum interleukin (IL)-6 or evening salivary cortisol concentrations predict posttraumatic stress disorder in children and adolescents six months after a motor vehicle accident. Psychoneuroendocrino. 2007;32(8-10):991-9.
